# Childhood Maltreatment, Pathological Personality Dimensions, and Suicide Risk in Young Adults

**DOI:** 10.3389/fpsyg.2018.00806

**Published:** 2018-05-23

**Authors:** Giorgio Falgares, Daniela Marchetti, Giovanna Manna, Pasquale Musso, Osmano Oasi, Daniel C. Kopala-Sibley, Sandro De Santis, Maria C. Verrocchio

**Affiliations:** ^1^Department of Psychological, Pedagogical and Educational Sciences, University of Palermo, Palermo, Italy; ^2^Department of Psychological, Health and Territorial Sciences, University of Chieti–Pescara, Chieti, Italy; ^3^Department of Languages and Educational Sciences, University of Calabria, Rende, Italy; ^4^Department of Psychology, Catholic University of Sacred Heart, Milan, Italy; ^5^Department of Psychiatry, Mathison Centre for Mental Health Research and Education, Hotchkiss Brain Institute, University of Calgary, Calgary, AB, Canada; ^6^Alberta Children’s Hospital Research Institute, University of Calgary, Calgary, AB, Canada; ^7^Azienda Provinciale peri Servizi Sanitari, Provincia Autonoma di Trento, Trento, Italy

**Keywords:** child maltreatment, personality traits, suicidal ideation, suicidal behavior, mediation effect

## Abstract

Several studies have demonstrated that child maltreatment (psychological, physical, and sexual abuse, and neglect) may be a significant factor in the development of pathological personality traits that increase the risk for suicidal ideation and behavior from adolescence to adulthood. Currently, the challenge is to understand how different forms of early negative experiences render an individual prone to develop specific personality traits and, in turn, be more vulnerable to suicide risk. To understand the relationship between childhood maltreatment and personality dimensions in suicide risk, our study aims to explore the role of self-criticism and dependency, two different pathological personality traits, as potential mediators of the link between different types of childhood maltreatment and suicide risk in young adults. For this purpose, 306 students from three Italian public universities were recruited. We used the Italian version of the Childhood Experience of Care and Abuse Questionnaire (CECA.Q) to assess experiences of lack of care by parents (i.e., antipathy and neglect) as well as psychological and physical abuse before the age of 17 years. The Depressive Experiences Questionnaire (DEQ) was used to assess the personality dimensions of self-criticism and dependency, and the Suicide History Self-Rating Screening Scale was administered to assess suicide risk. Results revealed that lack of care and psychological abuse were significantly associated with suicide risk and this association was partially mediated by the maladaptive personality dimension of self-criticism. These findings suggest that the combined effect of specific forms of dysfunctional parental behavior during childhood and the development of rigid and dysfunctional negative personality traits may increase the risk for suicidal ideation and behavior during adulthood.

## Introduction

Child maltreatment, such as physical and mental injury as well as sexual abuse and neglect, refers to acts of commission or omission by a parent or other caregiver that results in harm, or the threat of harm, to a child even if the harm is unintentional (Gilbert et al., 2009). These acts deprive children of the security and emotional support necessary for healthy development (Dube et al., 2003).

A large body of research has demonstrated that childhood maltreatment can lead to a range of adverse health outcomes later in life, such as depression, anxiety, substance abuse, and delinquent behavior (Bruffaerts et al., 2010; Brodsky and Biggs, 2012; Gershon et al., 2013).

Several studies have also documented that childhood maltreatment is an important risk factor for suicidal ideation and suicidal attempts (Enns et al., 2006; Brodsky and Stanley, 2008; Barbosa et al., 2014; Puzia et al., 2014; Afifi et al., 2016; Sachs-Ericsson et al., 2016). Recently, efforts have been made to understand the different impact of specific forms of maltreatment on adolescent and adult personality functioning (Witt et al., 2016) and, in turn, on risk for suicide (Dunn et al., 2013). Specifically, an important goal is to identify and understand the processes through which different forms of child maltreatment confer risk for suicidality later in life (Brent, 2011; Nock, 2012).

From this point of view, while several studies explored the mediating role of mental disorders, such as depression, anxiety, and borderline personality disorder (Anderson et al., 2002; Hahm et al., 2010; Gershon et al., 2013; Infurna et al., 2016a), relatively fewer studies have focused on the role of personality dimensions (e.g., low self-esteem and perfectionism) and how these can contribute to risk for suicidal behavior from adolescence to adulthood (Glassman et al., 2007; Campos and Holden, 2014).

Particularly, several studies have demonstrated that the pathological personality dimensions of self-criticism (Fazaa and Page, 2003; O’Connor, 2007; Campos and Mesquita, 2014; Falgares et al., 2017a) and dependency (Bornstein and O’Neill, 2000) are significant risk factors for suicide risk and are influenced by dysfunctional early relationships, such as those with parents (Blatt, 2004; Campos et al., 2013; Falgares et al., 2017a). However, to our knowledge, no study has specifically examined the links between childhood maltreatment, self-criticism and dependency, and risk for suicidality in adulthood. Accordingly, in this study we aimed to investigate the role of self-criticism and dependency as potential mediators of the relationship between different types of child maltreatment and suicide risk in young adults.

### The Association Among Developmental Adversities, Self-Criticism and Dependency

In Blatt’s (2008) two-configurations model, mature personality is a synergistic and balanced product of two main developmental dimensions: interpersonal relatedness, that is the capacity to be involved in intimate, mature, and mutually satisfactory relationships; and self-definition, involving the development of a realistic, integrated, and differentiated identity (see Luyten and Blatt, 2013; Kopala-Sibley and Zuroff, 2014, for reviews).

A delay or disruption in this normal developmental dialectic process could result in a rigid preoccupation with one of these two dimensions. An overemphasis on issues of relatedness is the basis of a pathological personality style that Blatt labeled *dependent/anaclitic*. In contrast, an overemphasis on issues of self-definition is the basis of the *self-critical/introjective* personality style (Blatt and Blass, 1996). According to Campos et al. (2014), a dependent personality style is characterized by intense feelings of loneliness, abandonment, helplessness, and weakness. Because of the lack of internalization of the experiences of care and affection or of the qualities of supportive and loving individuals, others are valued primarily for the care, and satisfaction they provide (Blatt, 1974, 2004). A self-critical personality style, in contrast, is characterized by feelings of unworthiness, inferiority, failure, and guilt, and involves a tendency to adopt a punitive self-stance once standards are not met (Blatt and Zuroff, 1992; Shahar and Priel, 2003).

Based on this theoretical framework, Blatt et al. (1976) developed the Depressive Experiences Questionnaire (DEQ). This self report questionnaire measures three orthogonal factors: dependency, self-criticism, and efficacy. Subsequently, Blatt et al. (1995) factor analyzed the construct of dependency and identified two facets. The first facet, dependence, involves a more immature tendency, including feelings of helplessness, fears, and apprehensions about separation and rejection. The second facet, relatedness, is characterized as more mature, and includes items that describe feelings of loneliness and loss in reaction to the end of a relationship with a particular individual. Other independent lines of research have similarly concluded that dependency has relatively more and less adaptive aspects (Rude and Burnham, 1995). It should be noted that self-criticism, dependence, and relatedness are not categorical personality types. They are conceptualized as continuous, nearly orthogonal dimensions of individual difference (Zuroff et al., 2004).

Some studies have found a connection between self-criticism and dependency and child maltreatment or poor-quality parenting. Campos et al. (2010) found that self-criticism mediated the relationship between early caretaking relationships with the mother and depressive symptoms. Neediness – a sub factor of dependency – mediated the relationship between early maternal overprotection and depressive symptoms. Some data show that emotional abuse, more than physical and sexual abuse, confers risk for the development of suicidal ideation (Puzia et al., 2014). On the contrary, other studies demonstrate that sexual and physical abuse are the strongest predictors of the initial development as well as the persistence of suicidal behavior (Bruffaerts et al., 2010). Lassri and Shahar (2012) suggested that self-criticism is an important mediator of the relationship between childhood emotional maltreatment and psychopathology in a group of undergraduate students. In the same way, but in a group of eating disorder patients, Speranza et al. (2005) found that self-criticism but not dependency is correlated with negative events or experiences in childhood. Baetens et al. (2015) highlighted that perceived parental expressed emotions have an important effect on adolescents’ well-being and non-suicidal self-injury (NSSI), with self-criticism mediating this relationship. An overview of the literature about this topic indicates that self-criticism is an important predictor of suicidality (Campos et al., 2013).

### The Association Among Self-Criticism, Dependency and Suicide Risk

The link among self-criticism, dependency and suicide risk has been investigated in several studies (see Falgares et al., 2017a for a summary). Fazaa and Page (2003) found that highly self-critical patients were more likely to have attempted suicide in response to a failure, and that their intent in attempting suicide was to escape from the actual events–expectations discrepancy. Dependent patients were more likely to have made their attempt in response to an interpersonal trigger which suggests that their intention in attempting suicide was to communicate their feelings of distress to others. Fazaa and Page (2009) also reported that adult participants with higher dependency showed higher rescue scores (i.e., using methods that made rescue more likely). In contrast, higher self-criticism was associated with a greater wish to die. Fehon et al. (2000) analyzed associations between dependency, self-criticism, impulsivity, and suicidal behavior in a sample of depressed hospitalized adolescents. They found that suicide risk did not significantly differ between highly self-critical and highly dependent patients. However, dependent individuals appeared generally to be more engaged in patterns of impulsive gestures and attempts, whereas self-critical individuals appeared less impulsive and more likely to plan acts of self-harm. Klomek et al. (2008), in a cross-sectional study, examined the relationship between suicidality and dependent and self-critical vulnerabilities among adolescents and found that suicidal participants have significantly higher levels of both self-criticism and dependency than non-suicidal inpatients and healthy controls. In a sample of adults, Campos et al. (2013) found that depressive symptoms mediated the association between self-critical perfectionism and suicidality. Highly self-critically perfectionistic individuals are vulnerable to intense depression, often accompanied by suicidal impulses, when confronted with stressful life events and, in particular, events that disrupt self-definition or a sense of personal achievement. Campos and Mesquita (2014) tested a model of suicidality that included dependency, self-criticism, anger-temperament, depression, and anger-in in a community of adolescents. Self-critical, dependent, and anger-in traits predicted depression, which in turn predicted suicidality directly and indirectly through anger-in. Finally, Campos and Holden (2014) in a Portuguese non-clinical sample of adults found that highly self-critical depressed individuals, were more likely to show help-seeking behaviors for emotional suffering (i.e., have visited a mental health professional) and were at greater risk for suicidal behaviors relative to less self-critical individuals.

Based on the above considerations, we aim to explore the mediating role of self-criticism and dependence/relatedness (the two factors of the dependent personality) in the relationship between different forms of child maltreatment and suicidal risk. Specifically, in line with previous studies that found that emotional maltreatment predicts negative attribution of the self (Pagura et al., 2006; Soffer et al., 2008), we hypothesized that antipathy and neglect, as well as psychological abuse, would be associated with higher levels of self-criticism. Furthermore, according to other studies (Grilo et al., 1999; Mendelson et al., 2002), we expected that physical and sexual abuse would be associated with higher levels of dependency. Finally, self-criticism is expected to be more strongly associated with suicide risk relative to dependency (Campos and Holden, 2014). It is important to note that some studies consider antipathy and neglect as one factor (Schimmenti and Bifulco, 2015). Following these studies, we chose to use the term “lack of care” to refer to these two forms of maltreatment.

## Materials and Methods

### Participants

Participants were drawn from a multisite undergraduate student data set (*N* = 306) collected at public universities located in northern (Lombardy), central (Abruzzo), and southern (Sicily) Italian regions. More than 96% of these students were attending psychology courses, characterized in Italy by a high female prevalence (more than 80%, see AlmaLaurea, 2017). Only a small proportion of them (1.63%) had missing information on one or more of the study variables. These participants were excluded from the analyses, given that the *p*-value for Little’s Missing Completely At Random test was not significant, χ^2^(19) = 16.27, *p* = 0.64, and because attrition analyses revealed no significant associations between demographic variables and missingness. We also excluded eight participants for having extreme outlier values after preliminary data processing. Our final sample thus consisted of 293 young adults (males = 16.72% and females = 83.28%, which closely reflects the gender distribution of the population of psychology students) aged 18–27 years (*M* = 21.57, *SD* = 2.02). Almost all of them were Caucasian Italians (95.22%), had no current occupation (92.83%), and were unmarried (97.27%). The majority came from middle-class backgrounds (80.20%), had married and cohabiting parents (81.91%) who had at least a high school education (71.33% for mothers and 64,85% for fathers). Nearly all participants indicated their birth mother (97.95%) and birth father (98.63%) as the reference figures who brought them up in childhood. About 11% had parental loss before 17 years of age (specifically, about 3.5% experienced death of mother or father and 7.5% parental separation). Only one (0.34%) had a previous hospitalization experience for psychological/psychiatric reasons, while about 16% had some psychological counseling with a psychologist or psychiatrist (including the freely accessible sessions provided by the university psychological counseling services), consisting of at least five meetings and usually for not more than 1 year.

### Measures

#### Socio-Demographics

Respondents were asked to indicate their gender, age, ethnicity, occupation, marital status, SES level, previous hospitalization experience, previous psychological counseling and its typology, as well as their parents’ marital status and education.

#### Childhood Experience of Care and Abuse Questionnaire

The Childhood Experience of Care and Abuse Questionnaire (CECA.Q; Bifulco et al., 2005) was used to retrospectively assess adverse childhood experiences before the age of 17. The CECA.Q was developed to mirror the corresponding interview measure (CECA; Bifulco et al., 1994) and showed good internal consistency in different contexts (e.g., Smith et al., 2002; Verrocchio et al., 2014; Infurna et al., 2016b). Accordingly, the translated Italian version of the CECA.Q used in this study was adapted by considering the validated Italian CECA interview (Giannone et al., 2011) and following the recommendations of the International Test Commission (2005). As suggested by the validation work of Bifulco et al. (2005), the Italian version of the questionnaire incorporates sections on parental loss, reference figures in childhood, and assesses parental care (antipathy and neglect), and parental psychological, physical, and sexual abuse.

Parental loss refers to either parental death or separation of 1 year or more due to a parent moving and permanently living elsewhere before age 17. These life events are important, considering, for example, that Agid et al. (1999) found that loss due to separation and loss due to death are both risk factors for mood disorders. Parental loss was assessed by asking two questions: (1) “Did either parent die before you were age 17?” and (2) “Have you ever been separated from your parent for 1 year or more before you were age 17,” both rated as 0 (*no*) and 1 (*yes*) for both the mother and the father. Three successive questions asked about the age, duration, and reason for separation from the parent(s). For this section, the score could range from 0 to 4, with higher scores indicating more severe loss during childhood. However, the distribution of the score was highly skewed (>3) due to a large number of observations at 0. To ensure an adequate sufficient distribution of cases for analysis (in practice, this usually means a minimum number of 10–20 cases per cell when considering each discrete value of the distribution, e.g., Warner, 2012, and Hahs-Vaughn, 2016), the variable was dichotomized (see MacCallum et al., 2002) with a score of 0 for absence of death of parents or separation from them for 1 year or more before age 17 and a score of 1 indicating the presence of at least one death of a parent or separation from one parent for 1 year or more before age 17.

Antipathy refers to hostility, coldness or rejection as well as ‘scapegoating’ behavior shown toward the child by parents or surrogate parents. The related subscale consists of eight paired items for maternal and paternal figures (sample item: “At times she/he made me feel I was a nuisance”). Items were rated on a 5-point Likert scale ranging from 1 (*no, not at all*) to 5 (*yes, definitely*) and were summed to create two indicators of maternal (α = 0.84) and paternal (α = 0.87) antipathy. In this study, we averaged these two variables to obtain a single indicator of parental antipathy (score ranging from 8 to 40, with higher scores indicating more parental antipathy during childhood).

Neglect refers to a distinct disinterest in the child’s material and physical care (e.g., food, clothing, and health), friendships, schoolwork, career prospects, and whereabouts. The related subscale consists of eight paired items for maternal and paternal figures (sample item: “She/he neglected my basic needs, e.g., food and clothes”). Items were rated on a 5-point Likert scale ranging from 1 (*no, not at all*) to 5 (*yes, definitely*) and were summed to create two indicators of maternal (α = 0.79) and paternal (α = 0.88) neglect. In this study, we averaged these two variables to obtain a single indicator of parental neglect (score ranging from 8 to 40, with higher scores indicating more parental neglect during childhood).

Psychological abuse refers to a highly controlling and domineering relationship of parental figures with the child, including humiliation, terrorization, cognitive disorientation, exploitation, and corruption or intentional deprivation of needs or valued objects. The range of such experiences and their frequency determine the severity of this form of abuse. The related subscale consists of 17 psychological abuse items paired with frequency items for maternal and paternal figures (sample item: “She/he liked to see me suffer” and “How Frequent?”). Items regarding the amount of psychological abuse were rated as 0 (*no*), 1 (*unsure*), or 2 (*yes*), while frequency items were rated as 0 (*never*), 1 (*once*), 2 (*rarely*), and 3 (*often*). In this study, each pair of items were multiplied, and the obtained scores were summed to create indicators of maternal (α = 0.85) and paternal (α = 0.83) psychological abuse. We averaged these two variables to obtain a single indicator of parental psychological abuse (score ranging from 0 to 102, with higher scores indicating more parental psychological abuse).

Physical abuse refers to violence toward the child by parents or other caregivers in the household, including attacks where implements such as belts or sticks are used, or punching or kicking occurs with the possibility of causing harm. The screening question for this section was “When you were a child or teenager, were you ever hit repeatedly with an implement (such as a belt or stick) or punched, kicked, or burnt by someone in the household?,” rated as 0 (*no*) and 1 (*yes*). Severity was determined by using four dichotomous items (0 = *no*, 1 = *yes*), measuring the intensity of the attack and its frequency (example item: “Did the hitting happen on more than one occasion?”). Thus, severity could range from 0 to 4, with higher scores indicating more severe physical abuse. We multiplied the obtained scores for each participant to obtain a single indicator of psychological abuse. However, because the distribution of this comprehensive score was highly skewed (>3) and to ensure an adequate distribution of cases for analysis, this variable was dichotomized with a score of 0 for absence of any episode of physical abuse and a score of 1 for presence of one or more of such episodes.

Sexual abuse refers to age-inappropriate physical contact or approach of a sexual nature by any adult to the child. Screening questions for this section were: (1) “When you were a child or teenager, did you ever have any unwanted sexual experiences?,” (2) “Did anyone force you or persuade you to have sexual intercourse against your wishes before age 17?,” and (3) “Can you think of any upsetting sexual experiences before age 17 with a related adult or someone in authority, e.g., teacher?” These questions were rated as 0 (*no*) and 1 (*yes*), with score ranging from 0 to 3 and higher scores indicating more sexual abuse during childhood. Severity was determined by using seven dichotomous items (0 = *no*, 1 = *yes*), measuring the degree and intrusiveness of sexual contact, the relationship of trust with the perpetrator, and the frequency and duration of the abuse (example items: “Was the other person someone you knew?,” “Did this person do it to you on more than one occasion?,” and “Did it involve sexual intercourse?”). Thus, severity could range from 0 to 7, with higher scores indicating more severe sexual abuse. We multiplied the obtained scores for each participant to obtain a single indicator of sexual abuse. However, because the distribution of this comprehensive score was highly skewed (>3) and to ensure an adequate distribution of cases for analysis, this variable was dichotomized with a score of zero for absence of any episode of sexual abuse and a score of one for presence of one or more of such episodes.

#### Depressive Experiences Questionnaire

The 66-item self-report Italian version of the DEQ (Blatt et al., 1976; Falgares et al., 2017b) was used to assess pathological personality traits. Despite its name, the DEQ measures stable personality traits which confer risk for the development of depression, but which do not measure acute depression (Mongrain and Zuroff, 1994; Zuroff et al., 2004). The DEQ yields factor scores for three primary factors: dependency, self-criticism, and efficacy. Within the DEQ dependency scale, it is possible to distinguish two subscales: dependence and relatedness (Blatt et al., 1995). For the purposes of this study, the third primary factor (i.e., efficacy) was not considered, while we used the two subscales of dependency, dependence (example item: “Without support from others who are close to me, I would be helpless”) and relatedness (example item: “I would feel like I’d be losing an important part of myself if I lost a very close friend”), and the self-criticism scale (example item: “I tend to be very critical of myself”). Items were scored on a 7-point Likert scale, ranging from 1 (*strongly disagree*) to 7 (*strongly agree*). Self-criticism was scored using the original factor scores of Blatt et al. (1976). Dependence and relatedness were scored using the scoring method of Blatt et al. (1995) by summing the items contained in the subscales (respectively, 10 and 8 items). The Italian version of the DEQ has good internal consistency and validity, similar to those of the original DEQ (Falgares et al., 2017b). In the present study, reliability as measured with the Cronbach’s α was 0.83 for self-criticism, 0.72 for dependence, and 0.79 for relatedness.

#### Suicidal History Self-Rating Screening Scale

The 16-item Suicidal History Self-Rating Screening Scale (SHSS; Innamorati et al., 2011) was used to assess propensity for suicide risk. Items were rated on a dichotomous scale (0 = *no*, 1 = *yes*). The SHSS assesses suicidal ideation (example item: “Have you ever thought about taking your own life?”), planning (example item: “Have you ever planned a way of taking your own life?”), or attempts (example item: “Have you ever tried to take your own life?”) both in the previous year and lifetime. Despite these different facets of suicide, a one-factor solution was obtained in the validation study of the scale (with a clinical sample), having a Kuder-Richardson-20 index of 0.95 and mean inter-item correlation of 0.53 (Innamorati et al., 2011). Another study with a non-clinical sample confirmed one-factor structure of the SHSS (Pompili et al., 2016). The SHSS has demonstrated good internal consistency and validity (Innamorati et al., 2011; Pompili et al., 2016). For these reasons, as well as due to analytical issues (see section “Descriptive Statistics and Preliminary Analyses”), a unitary score was used. Thus, items were summed to provide a score ranging between 0 and 16, with higher scores indicating higher propensity to suicide. For the present sample, Cronbach alpha was 0.87.

### Procedure

The local psychology department’s ethics committees approved this study and all procedures were performed in accordance with the Italian Association of Psychology (2015) ethical principles for psychological research, inspired by the Declaration of Helsinki and its revisions (World Medical Association, 2001) as well as by the American Psychological Association [APA]’s (2010) ethics guidelines. Data were collected during class time at three Italian universities (in the cities of Palermo, Chieti, and Milan). After informing participants about the purpose of the research, the voluntary nature of participation and the anonymity of responses, students in each class were asked to participate in the study. More than 95% agreed to participate and written informed consent was obtained from all of them prior to collecting data in groups of 25–30 students in a large space in order to ensure anonymity. Trained Italian researchers collected the data in each university. Participants could withdraw at any time. All students were told to call the psychology departments for any further information about the research.

### Statistical Analysis

We followed three main steps to conduct data analysis. First, we computed descriptive statistics for the key observed study variables including their prevalence in terms of moderate or marked levels as well as means and standard deviations, skewness and kurtosis indices, and minimum and maximum values of standardized scores. The latter descriptive analysis results allowed verification of the univariate normality of the distributions. When it was the case, non-normally distributed variables were transformed to improve normality and extreme outliers. Scores beyond | 3.29| standard deviations from the mean (Tabachnick and Fidell, 2013), were excluded from the analyses (not exceeding 5% of total participants including cases eliminated for having missing data). Furthermore, we used the Mahalanobis distance and the Mardia’s multivariate kurtosis coefficient to test the multivariate normality and identify other potential multivariate outliers. Then, the final descriptive statistics for the study variables were computed.

Second, we evaluated the associations of socio-demographic with the main study variables. Particularly, we conducted a multivariate analysis of variance (MANOVA) to examine whether participants’ scores for antipathy, neglect, psychological abuse, physical abuse, dependence, relatedness, self-criticism, and propensity for suicide differed based on region of university education (1 = North Italy, Lombardy; 2 = Central Italy, Abruzzo; 3 = South Italy, Sicily), gender (0 = male; 1 = female), SES (dummy coded: 0 = low-medium; 1 = medium-high); parents’ marital status (dummy coded: 0 = cohabiting; 1 = not cohabiting), parents’ education (dummy coded: 0 = at most one of parents with high school diploma; 1 = both parents with at least high school diploma), parental loss (as described in the section “Measures” this variable was dichotomized with 0 = no death of parents or separation from them for 1 year or more before age 17; 1 = at least death of one parent or separation from one parent for 1 year or more before age 17), and previous psychological counseling (0 = no; 1 = yes). We computed Pearson’s correlation coefficients to describe potential significant relations between age and the above-mentioned key study variables.

Third, after reporting bivariate correlations for the key study variables including the significant control demographic variables, we used structural equation modeling (SEM) to explore the mediating processes in the relation between different forms of childhood maltreatment and suicide risk in emerging adulthood. We initially presented the *a priori* model including the antecedent, mediating, outcome, and control variables as well as direct and indirect paths. Then, we carried out SEM analyses to test this model within *Mplus 7.2* (Muthén and Muthén, 2014) using the maximum likelihood estimation method. All indirect effects were tested using a Bootstrap sample of 1000 for the 95% confidence intervals. To evaluate the model fit we used multiple criteria (see Hu and Bentler, 1999; Kline, 2010; Faraci and Musso, 2013): the chi-square (χ^2^) statistic, the comparative fit index (CFI; Bentler, 1990), the Tucker-Lewis index (TLI; also called the non-normed fit index or NNFI, Bentler and Bonett, 1980), the root mean square error of approximation (RMSEA; Steiger, 1990) and its 90% confidence interval (CI) (Browne and Cudeck, 1993), and standardized root mean square residual (SRMR; Bentler, 2006). Based on results from a Monte Carlo analysis, Hu and Bentler (1999) suggested that a reasonably good fit is supported when CFI and TLI values are close to or greater than 0.95, RMSEA values are close to or less than 0.06, and SRMR values are close to or less than 0.08. For the RMSEA 90% CI, values below 0.05 for the lower bound and below 0.08 for the upper bound suggest acceptable fit (MacCallum et al., 1996).

## Results

### Descriptive Statistics and Preliminary Analyses

An initial data inspection showed that physical abuse, sexual abuse and the subscales of suicidal planning and suicidal attempts of SHSS presented a large number of observations equal to zero and were highly skewed (>3). This resulted in a not-positive definite covariance matrix. Thus, physical abuse and sexual abuse were dichotomized, while the three subscales of the SHSS were computed together to obtain a comprehensive score (for both choices, see section “Measures” for further details). **Tables 1, 2** summarize the descriptive statistics. Both the prevalence and the mean scores of the key study variables generally confirmed the non-clinical nature of the sample. Antipathy, neglect, psychological abuse, and propensity for suicide were not normally distributed (**Table 2**) with skewness and kurtosis values >|1.00| (Curran et al., 1996; Kline, 2010) as well as maximum values of standardized scores >3.29 (as dichotomous variables, physical abuse and sexual abuse were excluded from these considerations). For these reasons, a transformation was applied for non-normal variables by computing the square root for each distribution as the best solution. After re-calculating descriptive statistics for the transformed variables, eight cases presenting extreme outlier standardized scores (>|3.62|, calculated as 3.29 plus a tolerance of 10%) were excluded from the sample. The new distributions showed adequate skewness and kurtosis values (see **Table 2**). Because less than 10% of respondents indicated experiences of sexual abuse, we excluded this variable from subsequent analyses because this small number continued to preclude meaningful results. Using Mahalanobis distance with *p* < 0.001, no cases were identified as multivariate outliers. However, Mardia’s multivariate kurtosis coefficient slightly exceeded the critical value. We detected five potential multivariate outliers. After performing the subsequent analyses without or with these cases, we found no effect on the pattern of results. Thus, we retained these five cases in the final sample.

**Table 1:** Prevalence of the key study variables, calculated as the percentage of participants exceeding the cut-off scores for moderate or marked levels.

Observed variable	Cut-off scores for moderate or marked level	% (*N* = 301)
(1) Antipathy (scored 8–40)	≥25^a^	2.66
(2) Neglect (scored 8–40)	≥22^a^	6.64
(3) Psychological abuse (scored 0–102)	There is no established cut-off	–
(4) Physical abuse (scored 0 or 1)	≥1^a^	17.61
(5) Sexual abuse (scored 0 or 1)	≥1^a^	9.97
(6) Dependence (scored 10–70)	There is no established cut-off	–
(7) Relatedness (scored 8–56)	There is no established cut-off	–
(8) Self-criticism (factor derived scale score)	There is no established cut-off	–
(9) Propensity for suicide (scored 0–16)	≥8^b^	2.33
Suicidal ideation in the last year (scored 0 or 1)	≥1^b^	10.96
Suicidal planning in the last year (scored 0 or 1)	≥1^b^	5.65
Suicidal attempt in the last year (scored 0 or 1)	≥1^b^	0.66
More than one suicide attempt in the last year (scored 0 or 1)	≥1^b^	0.33
Suicidal ideation in the lifetime excluding the last year (scored 0 or 1)	≥1^b^	12.29
Suicidal planning in the lifetime excluding the last year (scored 0 or 1)	≥1^b^	6.98
Suicidal attempt in the lifetime excluding the last year (scored 0 or 1)	≥1^b^	2.33
More than one suicide attempt in the lifetime excluding the last year (scored 0 or 1)	≥1^b^	0.66

*^*a*^See Bifulco et al., 2005; ^*b*^See Innamorati et al., 2011.*

**Table 2:** Means, standard deviations, skewness, and kurtosis for the key study variables both in their original version and in their transformed version as well as including and excluding extreme outliers.

Observed variable	Transformation	*M*	*SD*	Skewness	Kurtosis	Min. stand.	Max. stand.
Initial sample, *N* = 301							
(1) Antipathy (scored 8–40)	No	14.48	5.17	1.17	1.51	-1.45	3.87
(2) Neglect (scored 8–40)	No	13.57	4.74	1.26	2.01	-1.18	4.20
(3) Psychological abuse (scored 0–102)	No	4.38	7.46	2.85	10.41	-0.59	6.12
(4) Physical abuse (scored 0 or 1)	No	0.18	0.38	1.71	0.93	-0.46	2.16
(5) Sexual abuse (scored 0 or 1)	No	0.10	0.30	2.75	5.60	-0.33	3.06
(6) Dependence (scored 10–70)	No	42.19	8.62	0.12	-0.01	-2.69	2.76
(7) Relatedness (scored 8–56)	No	37.47	7.27	-0.31	-0.25	-2.95	2.55
(8) Self-criticism (factor-derived scale score)	No	-0.15	1.43	-0.03	0.54	-2.80	3.70
(9) Propensity for suicide (scored 0–16)	No	1.21	2.22	2.25	5.50	-0.55	5.76
Final sample excluding extreme outliers, *N* = 293							
(1) Antipathy (scored 2.83–6.32)	Yes (square root)	3.71	0.60	0.59	-0.03	-1.77	3.53
(2) Neglect (scored 2.83–6.32)	Yes (square root)	3.61	0.58	0.72	0.43	-1.35	3.56
(3) Psychological abuse (scored 0–10.10)	Yes (square root)	1.30	1.49	0.89	-0.11	-0.87	3.29
(4) Physical abuse (scored 0 or 1)	No	0.16	0.37	1.83	1.34	-0.44	2.26
(5) Sexual abuse (scored 0 or 1)	No	0.10	0.29	2.77	5.69	-0.32	3.07
(6) Dependence (scored 10–70)	No	42.01	8.38	0.09	-0.07	-2.75	2.86
(7) Relatedness (scored 8–56)	No	37.40	7.08	-0.32	-0.29	-3.02	2.63
(8) Self-criticism (factor-derived scale score)	No	-0.18	1.37	-0.21	0.15	-2.90	2.94
(9) Propensity for suicide (scored 0–4)	Yes (square root)	0.60	0.89	1.19	0.18	-0.67	3.53

*Min. stand., minimum value of standardized score; Max. stand., maximum value of standardized score*.

We then examined the influence of the demographic variables (region of university education, gender, SES, parents’ marital status, parents’ education, parental loss, and previous psychological appointments) on the key study variables. The MANOVA resulted in a significant multivariate effect of gender, Wilks’ λ = 0.93, *F*(8,212) = 2.11, *p* = 0.036, η^2^ = 0.07. Univariate analyses of variance (ANOVAs) showed that gender had a significant effect on Dependence, *F*(1,219) = 4.23, *p* = 0.041, η^2^ = 0.02, and Relatedness, *F*(1,219) = 5.09, *p* = 0.025, η^2^ = 0.02. Females reported significantly higher levels of dependence and relatedness than did males. No significant multivariate effects were observed for region of university education, Wilks’ λ = 0.92, *F*(16,424) = 1.10, *p* = 0.35, η^2^ = 0.04, SES, Wilks’ λ = 0.95, *F*(8,212) = 1.46, *p* = 0.17, η^2^ = 0.05, parents’ marital status, Wilks’ λ = 0.99, *F*(8,212) = 0.22, *p* = 0.99, η^2^ = 0.01, parents’ education, Wilks’ λ = 0.98, *F*(8,212) = 0.60, *p* = 0.78, η^2^ = 0.02, parental loss, Wilks’ λ = 0.98, *F*(8,212) = 0.66, *p* = 0.72, η^2^ = 0.02, and previous psychological counseling, Wilks’ λ = 0.94, *F*(8,212) = 1.61, *p* = 0.12, η^2^ = 0.06. Furthermore, age was significantly and negatively related to dependence, *r* = -0.14, *p* = 0.015, and relatedness, *r* = -0.16, *p* = 0.007, but not to the other variables (see **Table 2**).

### SEM Analysis of the Mediation Model

Based on the preliminary analyses, gender and age were used as control variables. Correlations between the key and control study variables are displayed in **Table 3**.

**Table 3:** Correlations for key and control study variables.

		1	2	3	4	5	6	7	8	9	10
(1)	Antipathy	–									
(2)	Neglect	0.73***	–								
(3)	Psychological abuse	0.61***	0.56***	–							
(4)	Physical abuse	0.38***	0.32***	0.36***	–						
(5)	Dependence	0.22 ***	0.14*	0.24***	0.16***	–					
(6)	Relatedness	0.15*	0.05	0.15**	0.04	0.69***	–				
(7)	Self-criticism	0.48***	0.32***	0.42***	0.23***	0.30***	0.20***	–			
(8)	Propensity for suicide	0.53***	0.40***	0.54***	0.25***	0.24***	0.16**	0.48***	–		
(9)	Gender	-0.09	-0.15*	0.01	-0.02	0.18**	0.21***	-0.04	-0.10	–	
(10)	Age	0.07	0.05	-0.01	0.06	-0.14*	-0.16**	-0.07	-0.05	-0.09	–

*^∗^p < 0.05, ^∗∗^p < 0.01, ^∗∗∗^p < 0.001*.

**FIGURE 1:**
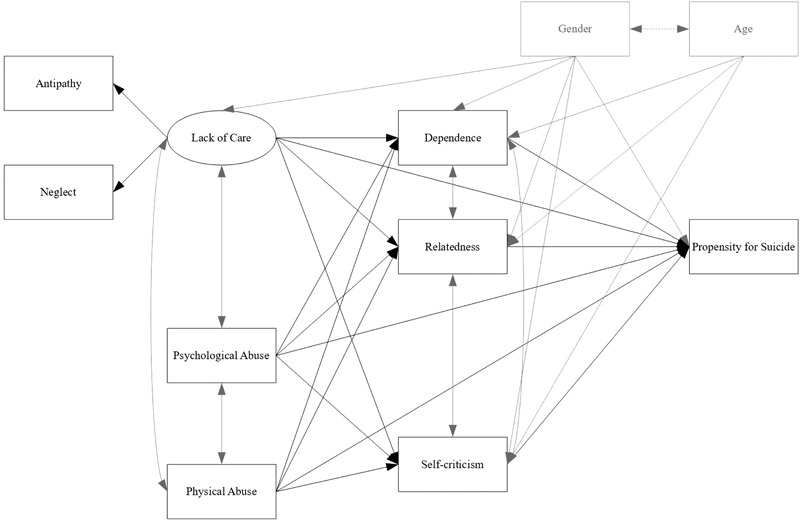
The theoretical model of the study. Covariances, control variables, and their related paths are presented in light gray.

To test our hypotheses, we used the *a priori* model presented in **Figure 1**. In view of our non-clinical sample, as suggested from literature (e.g., Bifulco et al., 2005) we considered antipathy and neglect as observed indicators of the latent variable lack of care. This variable as well as psychological abuse and physical abuse represented the antecedent variables influencing the outcome variable propensity for suicide, both directly and indirectly by the mediating role of dependence, relatedness and self-criticism. Gender was controlled for by allowing it to predict all the mediating and outcome variables as well as the latent variable lack of care (given the significant correlation with neglect, as shown in **Table 2**), while age was controlled by allowing it to predict only the mediating variables. The covariation within the antecedent variables, on one hand, and the mediating variables, on the other, was also allowed. This model had a good fit, χ^2^_SB_ (13) = 21.96, *p* = 0.06, CFI = 0.99, TLI = 0.97, RMSEA = 0.05, RMSEA 90% CI = 0.00–0.08, SRMR = 0.02.

**FIGURE 2:**
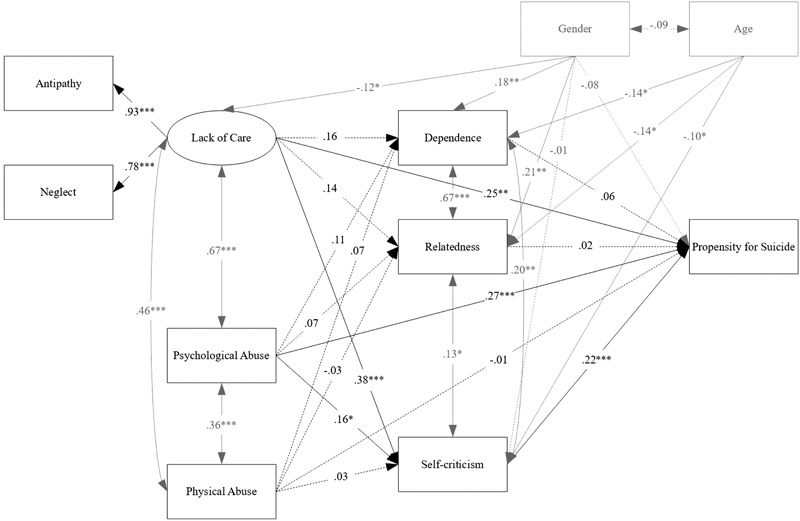
Estimated structural equation model. Maximum likelihood standardized coefficients are shown. Solid lines represent significant pathways, dashed lines are non-significant. Covariances, control variables and their related paths are presented in light gray. ^∗^*p* < 0.05, ^∗∗^*p* < 0.01, ^∗∗∗^*p* < 0.001.

Standardized coefficients are shown in **Figure 2**. Gender was significantly associated with lack of care as well as dependence and relatedness, such that experienced lower levels of lack of care and higher levels of dependence and relatedness than males. Age was significantly and negatively related to the mediating variables such that older participants showed lower levels of dependence, relatedness, and self-criticism. Over and above these effects, direct effects showed that lack of care (factor loadings linked to antipathy and neglect were adequate) and psychological abuse were significantly associated with propensity for suicide and higher levels of self-criticism, but not with dependence and relatedness. Self-criticism, but not dependence and relatedness, was significantly related to propensity for suicide. Physical abuse was not significantly linked to mediating or outcome variables. In summary, these results revealed that there was evidence of significant mediating effects of self-criticism in the relationships between lack of care, psychological abuse and suicidality. Specifically, while controlling for gender and age, lack of care, and psychological abuse were not only directly but also indirectly and positively related to propensity for suicide via self-criticism (respectively, β = 0.08, *p* = 0.002, 95% CI = 0.030–0.140, and β = 0.04, *p* = 0.047, 95% CI = 0.001–0.071).

## Discussion

The current study examined whether self-criticism and dependence/relatedness mediated the relationship between different forms of childhood maltreatment and suicide risk in young adults. We found an association between lack of care (i.e., antipathy and neglect), psychological abuse, and suicide risk. Furthermore, self-criticism partially mediated this effect. No association was found between physical abuse and suicide risk. Finally, neither dependence nor relatedness mediated the relationship between different types of maltreatment and suicide risk.

Our results are consistent with prior studies reporting high correlations among experiences of hostility, rejection, humiliation and parental criticism and the development of a representation of the self that is characterized by feelings of low self-worth, shame, guilt, and propensity to adopt a punitive self-stance, as a consequence of an internalization of a highly critical family environment (Soffer et al., 2008; Kopala-Sibley and Zuroff, 2014; Baetens et al., 2015; Verrocchio et al., 2015). In a recent meta-analysis, Liu et al. (2017) found that emotional abuse had the strongest effect on suicidal behavior compared to other types of maltreatment. Glassman et al. (2007) found that people who experienced maltreatment during childhood including repeated insults, excessive criticism, or some form of physical abuse, may develop a self-critical cognitive style over time through internalizing the behavior of those who criticized and abused them, which may ultimately result in NSSI behaviors.

Finally, other studies found a relationship between emotional maltreatment and neglect and suicide attempts after controlling for other forms of abuse (physical and sexual abuse) among adults (de Araujo and Lara, 2016; Springe et al., 2016).

In sum, these findings suggest that the combined effect of specific forms of dysfunctional parental behavior (e.g., parental criticism and a lack of emotional and material support) and the development of rigid and dysfunctional negative self-schemas (e.g., self-criticism), may increase suicide risk (Wedig and Nock, 2007; Yates et al., 2008; Claes et al., 2012).

Despite Blatt et al.’s (1995) suggestion that there are two facets of the DEQ dependency factor, namely dependence and relatedness results show that these facets of dependency did not mediate the relationship between different types of adversities and suicide risk. According to published data (Soffer et al., 2008; Lassri and Shahar, 2012) this result indicates that dependency may be a relatively less maladaptive factor than self-criticism in that it may not be linked to childhood maltreatment or suicide risk. However, we should note that this is somewhat in contrast to other studies which have found dependency to be influenced by early childhood and adolescent parenting and peer relationships (e.g., Kopala-Sibley et al., 2012; Kopala-Sibley et al., 2015).

Regarding gender differences, it is important to note that the prevalence of females in our sample may limit the generalizability of findings to males. Nevertheless, results are consistent with previous studies (Blatt and Blass, 1996; Kuperminc et al., 1997; Henrich et al., 2000), showing that females scored higher on dependency and self-criticism than males. Furthermore, consistent with other studies (Dube et al., 2003; Puzia et al., 2014; Sachs-Ericsson et al., 2016), results indicate that males scored higher than females on neglect and antipathy. Finally, we found that older males and females showed lower levels of self-criticism and dependency. Indeed, several studies suggest a normative improvement in one’s sense of relatedness and self-definition across the life span, as evidenced by age-related decreases in both self-criticism and dependency (Kopala-Sibley et al., 2013).

This study has several limitations. First, suicidality is linked to depression, although we did not measure depression in the current study. Thus, it is possible that symptoms of depression may account for some of the relationships found here, such as the association between self-criticism and suicidality. Second, like most research in this area, the present study is a cross-sectional investigation. This makes it impossible to document any causal relationship of child maltreatment and pathological personality traits with suicide risk. Third, all measures were self-reported, which might have inflated associations due to shared method variance. Moreover, childhood maltreatment was investigated retrospectively. Therefore, data did not necessarily reflect the full complement of omissive and commissive behaviors experienced by participants. Finally, a limitation involves the homogeneity of our sample of participants (all undergraduate-level students and enrolled in courses of Psychology, where there is a high female prevalence). A community sample would have increased the variability and strengthened the applicability of the results to the general population. This also raises the issue of whether college students might be an inappropriate comparison group against a clinical population, given age and other differences between these groups.

## Author Contributions

PM designed the computational framework for testing the model, analyzed the data, and contributed to the interpretation of the results. All authors participated in the concept and writing of this manuscript and approved the final version of the manuscript.

## Conflict of Interest Statement

The authors declare that the research was conducted in the absence of any commercial or financial relationships that could be construed as a potential conflict of interest.

## References

[B1] Afifi T. O. Taillieu T. Zamorski M. A. Turner S. Cheung K. Sareen J. (2016). Association of Child abuse exposure with suicidal ideation, suicide plans, and suicide attempts in military Personnel and the general population in Canada. 73 229–238. 10.1001/jamapsychiatry.2015.2732 26817953

[B2] Agid O. Shapira B. Zislin J. Ritsner M. Hanin B. Murad H. (1999). Environment and vulnerability to major psychiatric illness: a case control study of early parental loss in major depression, bipolar disorder and schizophrenia. 4 163–172. 10.1038/sj.mp.4000473 10208448

[B3] AlmaLaurea. (2017). Available at: http://www.almalaurea.it/sites/almalaurea.it/files/docs/universita/profilo/Profilo2016/profilo2016_04_caratteristiche_dei_laureati_al_momento_ingresso_universita.pdf

[B4] American Psychological Association [APA] (2010). Available at: http://www.apa.org/ethics/code/principles.pdf

[B5] Anderson P. L. Tiro J. A. Price A. W. Bender M. A. Kaslow N. J. (2002). Additive impact of childhood emotional, physical, and sexual abuse on suicide attempts among low-income African American Women. 32 131–138. 10.1521/suli.32.2.131.24405 12079029

[B6] Baetens I. Claes L. Hasking P. Smits D. Grietens H. Onghena P. (2015). The relationship between parental expressed emotions and non-suicidal self-injury: the mediating roles of self-criticism and depression. 24 491–498. 10.1007/s10826-013-9861-8

[B7] Barbosa L. P. Quevedo L. da Silva Gdel G. Jansen K. Pinheiro R. T. Branco J. (2014). Childhood trauma and suicide risk in a sample of young individuals aged 14-35 years in southern Brazil. 38 1191–1196. 10.1016/j.chiabu.2014.02.008 24629481

[B8] Bentler P. M. (1990). Comparative fit indexes in structural models. 107 238–246. 10.1037/0033-2909.107.2.238 2320703

[B9] Bentler P. M. (2006). Encino, CA: Multivariate Software, Inc.

[B10] Bentler P. M. Bonett D. G. (1980). Significance tests and goodness of fit in the analysis of covariance structures. 88 588–606. 10.1037/0033-2909.88.3.588

[B11] Bifulco A. Bernazzani O. Moran P. M. Jacobs C. (2005). The childhood experience of care and abuse questionnaire (CECA.Q): validation in a community series. 44 563–581. 10.1348/014466505X35344 16368034

[B12] Bifulco A. Brown G. W. Harris T. O. (1994). Childhood experience of care and abuse (CECA): a retrospective interview measure. 35 1419–1435. 10.1111/j.1469-7610.1994.tb01284.x 7868637

[B13] Blatt S. J. (1974). Levels of object representation in anaclitic and introjective depression. 29 107–157. 10.1080/00797308.1974.11822616 4445397

[B14] Blatt S. J. (2004). Washington, DC: American Psychological Association 10.1037/10749-000

[B15] Blatt S. J. (2008). Washington, DC: American Psychological Association.

[B16] Blatt S. J. Blass R. B. (1996). “Relatedness and self-definition: a dialectic model of personality development,” in ed. Noam G. G. (Hillsdale, NJ: Erlbaum) 309–338.

[B17] Blatt S. J. D’Afflitti J. P. Quinlan D. M. (1976). Experiences of depression in normal young adults. 85 383–389. 10.1037//0021-843X.85.4.383 956505

[B18] Blatt S. J. Zohar A. H. Quinlan D. M. Zuroff D. C. Mongrain M. (1995). Subscales within the dependency factor of the Depressive Experiences Questionnaire. 64 319–339. 10.1207/s15327752jpa6402 7722857

[B19] Blatt S. J. Zuroff D. C. (1992). Interpersonal relatedness and self-definition: two prototypes for depression. 12 527–562. 10.1016/0272-7358(92)90070-O

[B20] Bornstein R. O’Neill R. M. (2000). Dependency and suicidality in psychiatric inpatients. 56 463–473. 10.1002/(SICI)1097-4679(200004)56:4<463::AID-JCLP2>3.0.CO;2-5 10775041

[B21] Brent D. A. (2011). Preventing youth suicide: time to ask how. 50 738–740. 10.1016/j.jaac.2010.09.017 21784291

[B22] Brodsky B. S. Biggs E. (2012). Adverse childhood experiences and suicidal behavior. 17 16–21. 10.1016/j.psc.2008.02.002 18439446

[B23] Brodsky B. S. Stanley B. (2008). Adverse childhood experiences and suicidal behavior. 31 223–235. 10.1016/j.psc.2008.02.002 18439446

[B24] Browne M. W. Cudeck R. (1993). “Alternative ways of assessing model fit,” in eds Bollen K. A. Long J. S. (Newbury Park, CA: Sage) 136–162.

[B25] Bruffaerts R. Demyttenaere K. Borges G. Haro J. M. Chiu W. T. Hwang I. (2010). Childhood adversities as risk factors for onset and persistence of suicidal behaviour. 197 20–27. 10.1192/bjp.bp.109.074716 20592429 PMC2894980

[B26] Campos R. Besser A. Abreu H. Parreira T. Blatt S. (2014). Personality vulnerabilities in adolescent suicidality: the mediating role of psychological distress. 78 115–139. 10.1521/bumc.2014.78.2.115 24870846

[B27] Campos R. C. Besser A. Blatt S. J. (2010). The mediating role of self-criticism and dependency in the association between perceptions of maternal caring and depressive. 27 1149–1157. 10.1002/da.20763 21132848

[B28] Campos R. C. Besser A. Blatt S. J. (2013). Recollections of parental rejection, self-criticism and depression in suicidality. 17 58–74. 10.1080/13811118.2013.748416 23387404

[B29] Campos R. C. Holden R. R. (2014). Suicide risk in a Portuguese non-clinical sample of adults. 28 230–241. 10.4321/S0213-61632014000400004

[B30] Campos R. C. Mesquita C. (2014). Testing a model of suicidality in community adolescents: a brief report. 147 34–56. 10.4172/2375-4494.1000147

[B31] Claes L. Soenens B. Vansteenkiste M. Vandereycken W. (2012). The scars of the inner critic: Perfectionism and nonsuicidal self-injury in eating disorders. 20 196–202. 10.1002/erv.1158 21915946

[B32] Curran P. J. West S. G. Finch J. F. (1996). The robustness of test statistics to nonnormality and specification error in confirmatory factor analysis. 1 16–29. 10.1037/1082-989X.1.1.16

[B33] de Araujo R. M. Lara D. R. (2016). More than words: the association of childhood emotional abuse and suicidal behavior. 37 14–21. 10.1016/j.eurpsy.2016.04.002 27442978

[B34] Dube S. R. Felitti V. J. Dong M. Chapman D. P. Giles W. H. Anda R. F. (2003). Childhood abuse, neglect, and household dysfunction and the risk of illicit drug use: the adverse childhood experiences study. 111 564–572. 10.1542/peds.111.3.564 12612237

[B35] Dunn E. C. McLaughlin K. A. Slopen N. Rosand J. Smoller J. W. (2013). Developmental timing of child maltreatment and symptoms of depression and suicidal ideation in young adulthood: results from the National Longitudinal Study on Adolescent Health. 30 1–15. 10.1002/da.22102 23592532 PMC3873604

[B36] Enns M. W. Cox B. J. Afifi T. O. De Graaf R. Ten Have M. Sareen J. (2006). Childhood adversities and risk for suicidal ideation and attempts: a longitudinal population-based study. 36 1–10. 10.1017/S0033291706008646 16999880

[B37] Falgares G. Marchetti D. De Santis S. Carrozzino D. Kopala-Sibley D. C. Fulcheri M. (2017a). Attachment styles and suicide-related behaviors in adolescence: the mediating role of self-criticism and dependency. 8:36. 10.3389/fpsyt.2017.00036 28344562 PMC5344916

[B38] Falgares G. De Santis S. Gullo S. Carrozzino D. Marchetti D. Verrocchio M. C. (2017b). The Italian version of the depressive experiences questionnaire: psychometric properties and validation in students, community, and clinical groups. 20 81–90. 10.4081/ripppo.2017.227 PMC7451319 32913730

[B39] Faraci P. Musso P. (2013). “La valutazione dei modelli di equazioni strutturali [The evaluation of structural equation models],” in eds Barbaranelli C. Ingoglia S. (Milano, IT: LED) 111–150. 10.7359/649-2013-fara

[B40] Fazaa N. Page S. (2003). Dependency and self-criticism as predictors of suicidal behavior. 33 172–185. 10.1521/suli.33.2.172.22777 12882418

[B41] Fazaa N. Page S. (2009). Personality style and impulsivity as determinants of suicidal subgroups. 13 31–45. 10.1080/13811110802572122 19123107

[B42] Fehon D. C. Grilo C. M. Martino S. (2000). A comparison of dependent and self-critically depressed hospitalized adolescents. 29 93–106. 10.1023/A:1005125322629

[B43] Gershon A. Sudheimer K. Tirouvanziam R. Williams L. M. O’Hara R. (2013). The long-term impact of early adversity on late-life psychiatric disorders. 15:352. 10.1007/s11920-013-0352-9 23443532

[B44] Giannone F. Schimmenti A. Caretti V. Chiarenza A. Ferraro A. Guarino S. (2011). Validità, attendibilità e proprietà psicometriche della versione Italiana dell’intervista CECA (Childhood Experience of Care and Abuse).[Validity, reliability and psychometric properties of the Italian translation of the CECA interview (Childhood experience of care and abuse)]. 30 3–21.

[B45] Gilbert R. Widom C. S. Browne K. Fergusson D. Webb E. Janson J. (2009). Burden and consequences of child maltreatment in high-income countries. 373 68–81. 10.1016/S0140-6736(08)61706-7 19056114

[B46] Glassman L. H. Weierich M. R. Hooley J. M. Deliberto T. L. Nock M. K. (2007). Child maltreatment, non-suicidal self-injury, and the mediating role of self-criticism. 45 2483–2490. 10.1016/j.brat.2007.04.002 17531192 PMC2034449

[B47] Grilo C. M. Sanislow C. Fehon D. C. Martino S. McGlashan T. H. (1999). Psychological and behavioral functioning in adolescent psychiatric inpatients who report histories of childhood abuse. 156 538–543. 10.1176/ajp.156.4.538 10200731

[B48] Hahm H. C. Lee Y. Ozonoff A. Van Wert M. J. (2010). The impact of multiple types of child maltreatment on subsequent risk behaviors among women during the transition from adolescence to young adulthood. 39 528–540. 10.1007/s10964-009-9490-0 20020190 PMC2850954

[B49] Hahs-Vaughn D. L. (2016). New York, NY: Taylor & Francis.

[B50] Henrich C. C. Kuperminc G. P. Sack A. Blatt S. J. Leadbeater B. J. (2000). Characteristics and homogeneity of early adolescent friendship groups: A comparison of male and female cliques and nonclique members. 4 15–26. 10.1207/S1532480XADS0401_2

[B51] Hu L. Bentler P. M. (1999). Cutoff criteria for fit indexes in covariance structure analysis: Conventional criteria versus new alternatives. 6 1–55. 10.1080/10705519909540118

[B52] Infurna M. R. Brunner R. Holz B. Parzer P. Giannone F. Reichl C. (2016a). The specific role of childhood abuse, parental bonding, and family functioning in female adolescents with borderline personality disorder. 30 177–192. 10.1521/pedi_2015_29_186 25905734

[B53] Infurna M. R. Reichl C. Parzer P. Schimmenti A. Bifulco A. Kaess M. (2016b). Associations between depression and specific childhood experiences of abuse and neglect: a meta-analysis. 190 47–55. 10.1016/j.jad.2015.09.006 26480211

[B54] Innamorati M. Pompili M. Serafini G. Lester D. Erbuto D. Amore M. (2011). Psychometric properties of the suicidal history self-rating screening scale. 15 87–92. 10.1080/13811118.2011.540471 21294003

[B55] International Test Commission (2005). Available at: https://www.intestcom.org/files/guideline_test_adaptation.pdf

[B56] Italian Association of Psychology. (2015). Available at: http://www.aipass.org/node/11560

[B57] Kline R. B. (2010). 3rd Edn. New York, NY: Guilford Press.

[B58] Klomek A. Orbach I. Sher L. Sommerfeld E. Diller R. Apter A. (2008). Quality of depression among suicidal inpatient youth. 12 133–140. 10.1080/13811110701857160 18340595

[B59] Kopala-Sibley D. C. Mongrain M. Zuroff D. C. (2013). A lifespan perspective on dependency and self-criticism: age-related differences from 18 to 59. 20 126–141. 10.1007/s10804-013-9163-9

[B60] Kopala-Sibley D. C. Zuroff D. C. (2014). The developmental origins of personality factors from the self-definitional and relatedness domains: a review of theory and research. 18 137–155. 10.1037/gpr0000013

[B61] Kopala-Sibley D. C. Zuroff D. C. Hankin B. L. Abela J. R. (2015). The development of self-criticism and dependency in early adolescence and their role in the development of depressive and anxiety symptoms. 41 1094–1109. 10.1177/0146167215590985 26091911

[B62] Kopala-Sibley D. C. Zuroff D. C. Leybman M. J. Hope N. (2012). The developmental origins of dependency-related vulnerabilities to depression: recalled peer attachments and current levels of neediness and connectedness. 44 264–271. 10.1037/a0028952

[B63] Kuperminc G. P. Blatt S. J. Leadbeater B. J. (1997). Relatedness, self-definition, and early adolescent adjustment. 21 301–320. 10.1023/A:1021826500037

[B64] Lassri D. Shahar G. (2012). Self-criticism mediates the link between childhood emotional maltreatment and young adults’ romantic relationships. 31 289–311. 10.1521/jscp.2012.31.3.289

[B65] Liu J. Fang Y. Gong J. Cui X. Meng T. Xiao B. (2017). Associations between suicidal behavior and childhood abuse and neglect: a meta-analysis. 220 147–155. 10.1016/j.jad.2017.03.060 28623759

[B66] Luyten P. Blatt S. J. (2013). Interpersonal relatedness and self-definition in normal and disrupted personality development: retrospect and prospect. 68 172–183. 10.1037/a0032243 23586492

[B67] MacCallum R. C. Browne M. W. Sugawara H. M. (1996). Power analysis and determination of sample size in covariance structure modeling. 1 130–149. 10.1037/1082-989X.1.2.130

[B68] MacCallum R. C. Zhang S. Preacher K. J. Rucker D. D. (2002). On the practice of dichotomization of quantitative variables. 7 19–40. 10.1037//1082-989X.7.1.19 11928888

[B69] Mendelson T. Robins C. L. Johnson C. S. (2002). Relations of sociotropy and autonomy to developmental experiences among psychiatric patients. 26 189–198. 10.1023/A:1014569703020

[B70] Mongrain M. Zuroff D. C. (1994). Ambivalence over emotional expression and negative life events: Mediators of depressive symptoms in dependent and self-critical individuals. 16 447–458. 10.1016/0191-8869(94)90071-X

[B71] Muthén B. O. Muthén L. K. (2014). Los Angeles, CA: Muthén & Muthén.

[B72] Nock M. K. (2012). Future directions for the study of suicide and self-injury. 41 255–259. 10.1080/15374416.2012.652001 22417198

[B73] O’Connor R. C. (2007). The relations between perfectionism and suicidality: a systematic review. 37 698–714. 10.1521/suli.2007.37.6.698 18275376

[B74] Pagura J. Cox B. J. Sareen J. Enns M. W. (2006). Childhood adversities associated with self-criticism in a nationally representative sample. 41 1287–1298. 10.1016/j.paid.2006.05.003

[B75] Pompili M. Innamorati M. Lamis D. A. Lester D. Di Fiore E. Giordano G. (2016). The interplay between suicide risk, cognitive vulnerability, subjective happiness and depression among students. 35 450–458. 10.1007/s12144-015-9313-2

[B76] Puzia M. E. Kraines M. A. Liu R. T. Kleiman E. M. (2014). Early life stressors and suicidal ideation: mediation by interpersonal risk factors. 56 68–72. 10.1016/j.paid.2013.08.027

[B77] Rude S. S. Burnham B. L. (1995). Connectedness and neediness: factors of the DEQ and SAS dependency scales. 19 323–340. 10.1007/BF02230403

[B78] Sachs-Ericsson N. J. Rushing N. C. Stanley I. H. Sheffler J. (2016). In my end in my beginning: developmental trajectories of adverse childhood experiences to late-life suicide. 20 139–165. 10.1080/13607863.2015.1063107 26264208

[B79] Schimmenti A. Bifulco A. (2015). Linking lack of care in childhood to anxiety disorders in emerging adulthood: the role of attachment styles. 20 41–48. 10.1111/camh.12051 32680332

[B80] Shahar G. Priel B. (2003). Active vulnerability, adolescent distress, and the mediating/suppressing role of life events. 35 199–218. 10.1016/S0191-8869(02)00185-X

[B81] Smith N. Lam D. Bifulco A. Checkley S. (2002). Childhood experience of care and abuse questionnaire (CECA.Q). 37 572–579. 10.1007/s00127-002-0589-9 12545234

[B82] Soffer N. Gilboa–Schechtman E. Shahar G. (2008). The relationship of childhood emotional abuse and neglect to depressive vulnerability and low self-efficacy. 1 151–162. 10.1521/ijct.2008.1.2.151

[B83] Speranza M. Corso M. Loas G. Stéphan P. Guilbaud O. Perez-Diaz F. (2005). Depressive personality dimensions and alexithymia in eating disorders. 135 153–163. 10.1016/j.psychres.2005.04.001 15913785

[B84] Springe L. Pulmanis T. Velika B. Pudule I. Grinberga D. Villerusa A. (2016). Self-reported suicide attempts and exposure to different types of violence and neglect during childhood: Findings from a young adult population survey in Latvia. 44 411–417. 10.1177/1403494816631394 26865568

[B85] Steiger J. S. (1990). Structural model evaluation and modification: an interval estimation approach. 25 173–180. 10.1207/s15327906mbr2502_4 26794479

[B86] Tabachnick B. G. Fidell L. S. (2013). 6th Edn. Boston, MA: Allyn and Bacon.

[B87] Verrocchio M. C. Marchetti D. Baker A. J. (2014). Assessment of psychological maltreatment: psychometric properties of the psychological maltreatment measure – Italian version. 16 57–75. 10.3280/MAL2014-001004

[B88] Verrocchio M. C. Marchetti D. Fulcheri M. (2015). Perceived parental functioning, self-esteem, and psychological distress in adults whose parents are separated/divorced. 6:1760. 10.3389/fpsyg.2015.01760 26635670 PMC4646956

[B89] Warner R. M. (2012). Thousand Oaks, CA: Sage.

[B90] Wedig M. M. Nock M. K. (2007). Parental expressed emotion and adolescent self-injury. 46 1171–1178. 10.1097/chi.0b013e3180ca9aaf 17712240

[B91] Witt A. Münzer A. Ganser H. G. Fegert J. M. Goldbeck L. Plener P. L. (2016). Experience by children and adolescents of more than one type of maltreatment: association of different classes of maltreatment profiles with clinical outcome variables. 57 1–11. 10.1016/j.chiabu.2016.05.001 27254375

[B92] World Medical Association (2001). Declaration of Helsinki. Ethical principles for medical research involving human subjects. 79 373–374. PMC2566407 11357217

[B93] Yates T. M. Tracy A. J. Luthar S. S. (2008). Nonsuicidal self-injury among “privileged” youths: longitudinal and cross-sectional approaches to developmental process. 76 52–62. 10.1037/0022-006X.76.1.52 18229983 PMC4354956

[B94] Zuroff D. C. Mongrain M. Santor D. A. (2004). Conceptualizing and measuring personality vulnerability to depression: comment on Coyne and Whiffen (1995). 130 489–511. 10.1037/0033-2909.130.3.489 15122935

